# Supplementary material for: Gene networks that compensate for crosstalk with crosstalk
Source: Nat Commun. 2019 Sep 6;10:4028. doi: 10.1038/s41467-019-12021-y (PMC6731275; doi:10.1038/s41467-019-12021-y)
Supplement: Supplementary file 1 — Supplementary Information [file 41467_2019_12021_MOESM1_ESM.pdf]

# Gene Networks that Compensate for Crosstalk With Crosstalk

Müller et al.

A

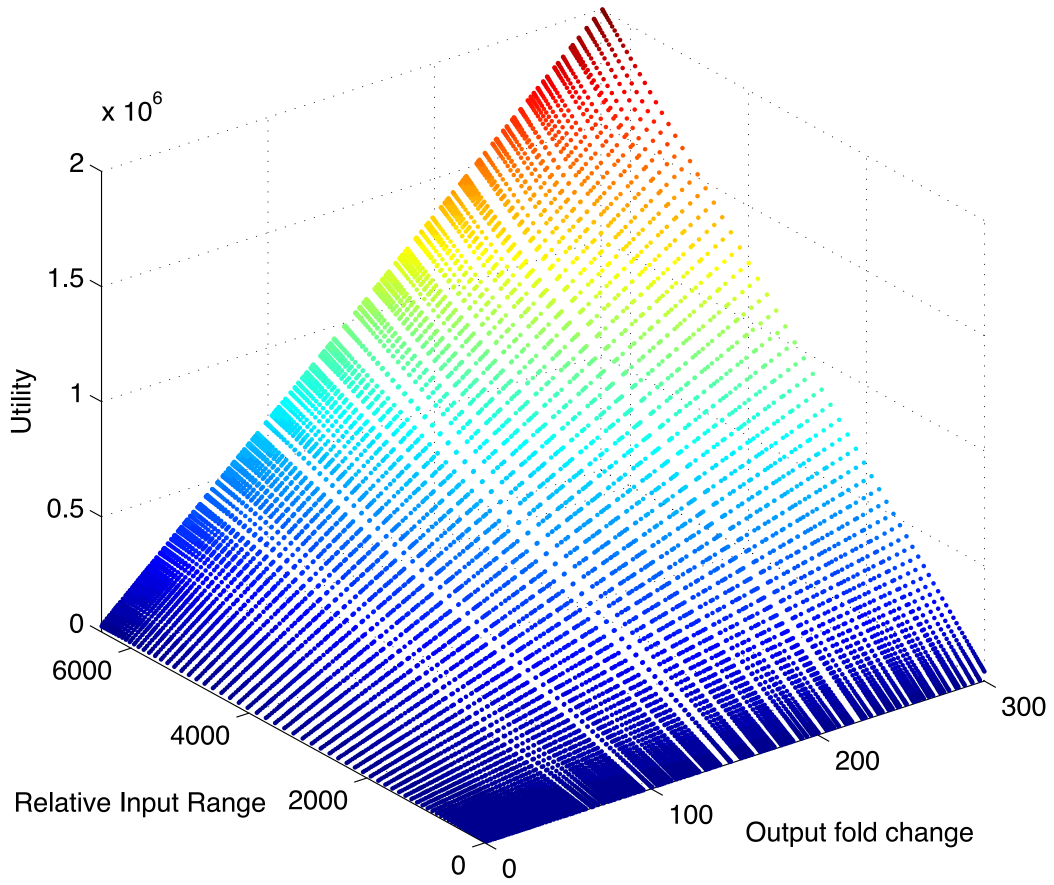
x 10^6
2
1.5
1
0.5
0
Utility
6000
4000
2000
0
Relative Input Range
0
100
200
300
Output fold change

B

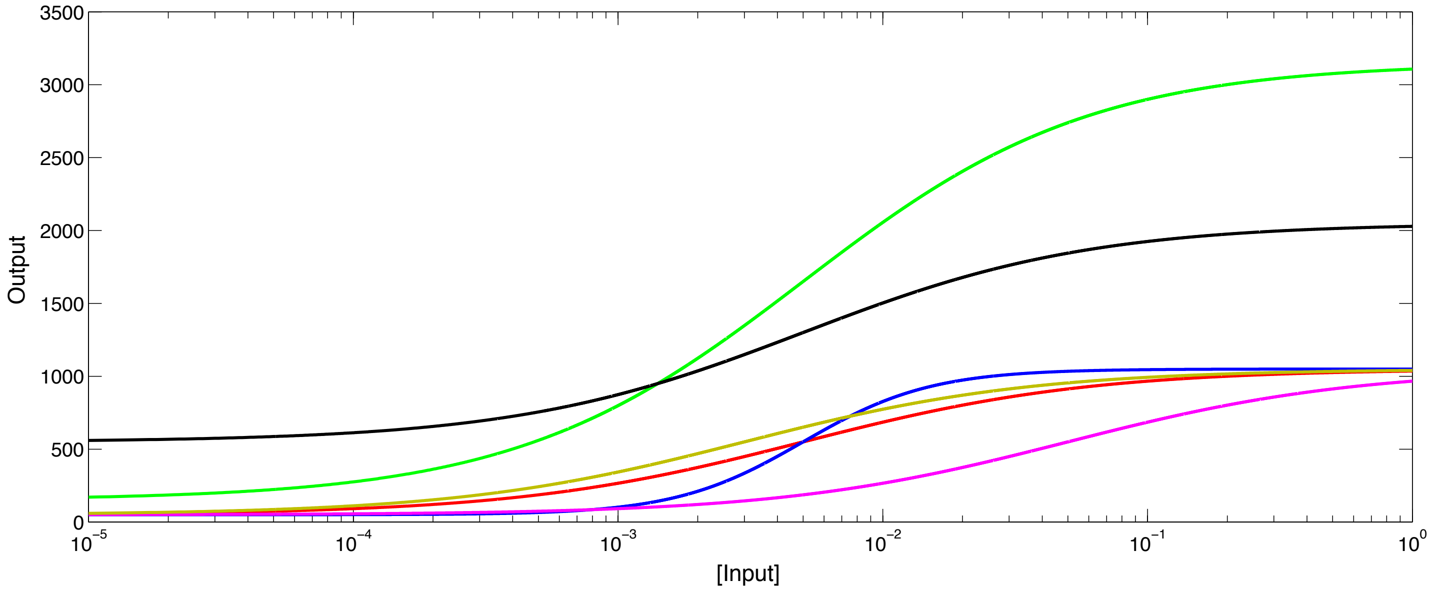
3500
3000
2500
2000
1500
1000
500
0
Output
10^-5
10^-4
10^-3
10^-2
10^-1
10^0
[Input]

C

| | 1) Red | 2) Blue | 3) Green | 4) Black | 5) Purple | 6) Yellow |
|---|---|---|---|---|---|---|
| **bmax** | 1000 | 1000 | 3000 | 1500 | 1000 | 1000 |
| **C** | 50 | 50 | 150 | 550 | 50 | 50 |
| **$K_d$** | 0.005 | 0.005 | 0.005 | 0.005 | 0.05 | 0.003 |
| **n** | 0.8 | 1.8 | 0.8 | 0.8 | 0.8 | 0.8 |
| **Output Fold Induction** | 21 | 21 | 21 | 3.73 | 21 | 21 |
| **Relative Input Range** | 243 | 11.49 | 243 | 243 | 243 | 243 |
| **Utility** | 5103 | 241.29 | 5103 | 906.39 | 5103 | 5103 |

**Supplementary Figure 1 | The behavior of the utility metric with different parameters in hypothetical Hill curves. (A)** The utility metric was simulated in Matlab over a range of *Bmax*, *C,* and *n* values. *Bmax* ranged from 1000 to 30256.5. *C* ranged from 100.2 to 1000.7. *n* ranged from .5 to 1.5. **(B)** Hill equations with shifted basal and maximum gene expression, $K_d$, and *n* were simulated in Matlab and **(C)** their utility values were calculated. The red and blue functions differ in the Hill coefficient, *n*. The red and green functions differ in their *C* and *bmax* values but have the same output fold-induction and same utility. The black function is shifted vertically relative to the red function, giving the same difference between *bmax* and *C*, but a lower output fold-induction and utility. The yellow function is shifted to lower $K_d$ relative to the red function, and the purple function is shifted to a higher $K_d$ relative to the red function, but all three functions have the same utility.

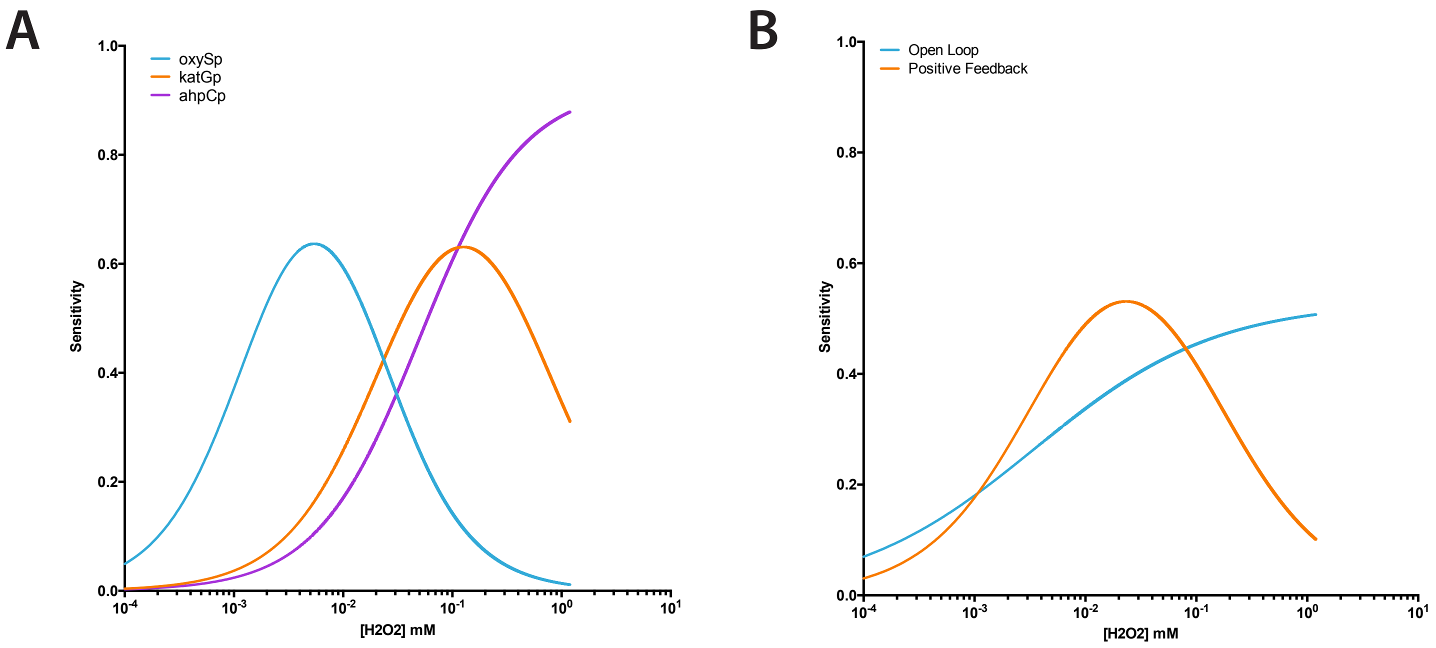


**Supplementary Figure 2 | Sensitivities for different $H_2O_2$-sensing circuits. (A)** The sensitivity of the circuits in Fig. 1A was calculated from the Hill equations plotted in Fig. 1B. **(B)** The sensitivity of the circuits in Fig. 1D and 1E was calculated from the Hill equations plotted in Fig. 1F. The sensitivity was calculated as in (1)**:**

$$S(x) = \frac{\frac{\partial y}{y}}{\frac{\partial x}{x}}$$

A

B

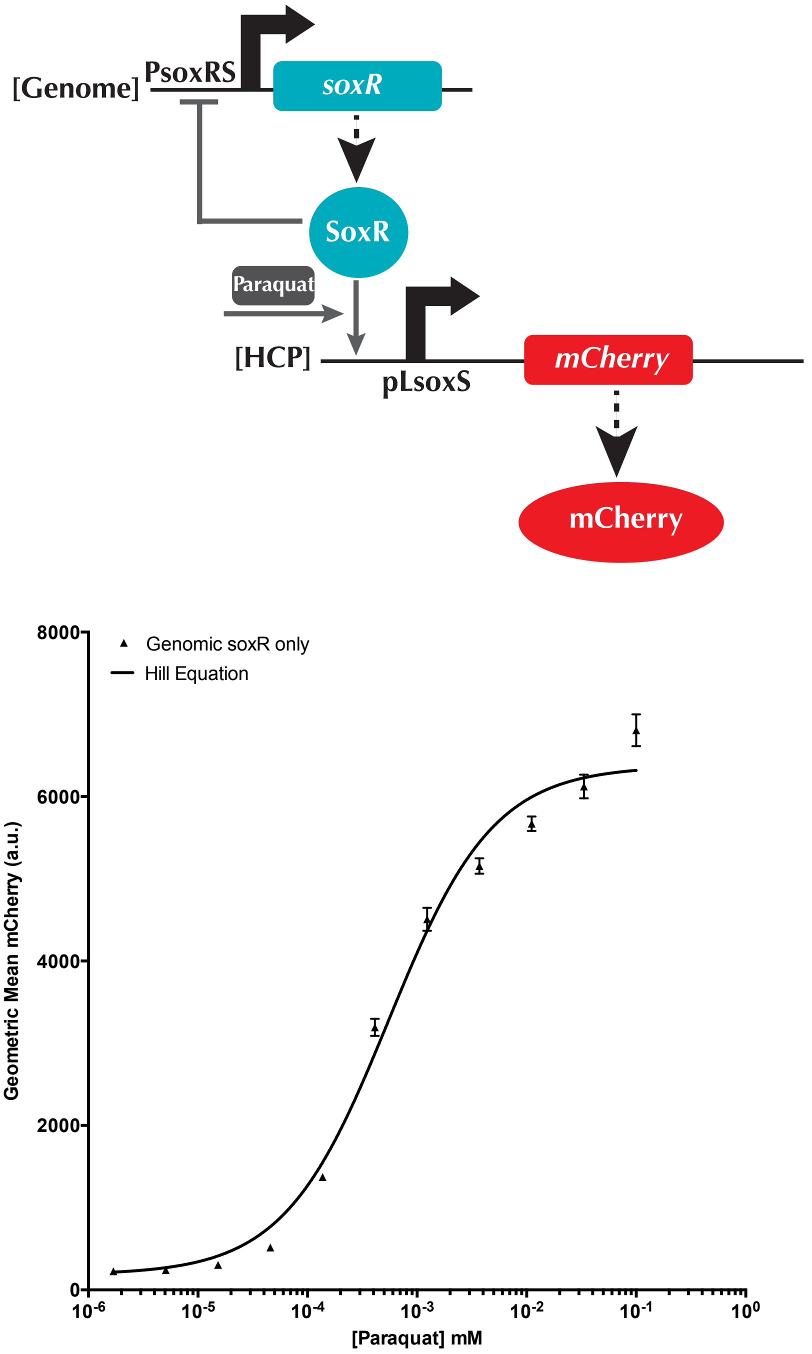


**Supplementary Figure 3 | Genomic *soxR* circuit for paraquat sensing. (A)** The genomic *soxR* circuit. *SoxR* is expressed from the genome and negatively regulates its own expression (2). *mCherry* expression is controlled by the pLsoxS promoter on a HCP. **(B)** The empirical paraquat-mCherry transfer function for the genomic soxR circuit. The line is a Hill function fit. The errors (s.e.m.) are derived from three biological replicates and flow cytometry experiments, each involving 30,000 events. Source data are provided as a Source Data file.

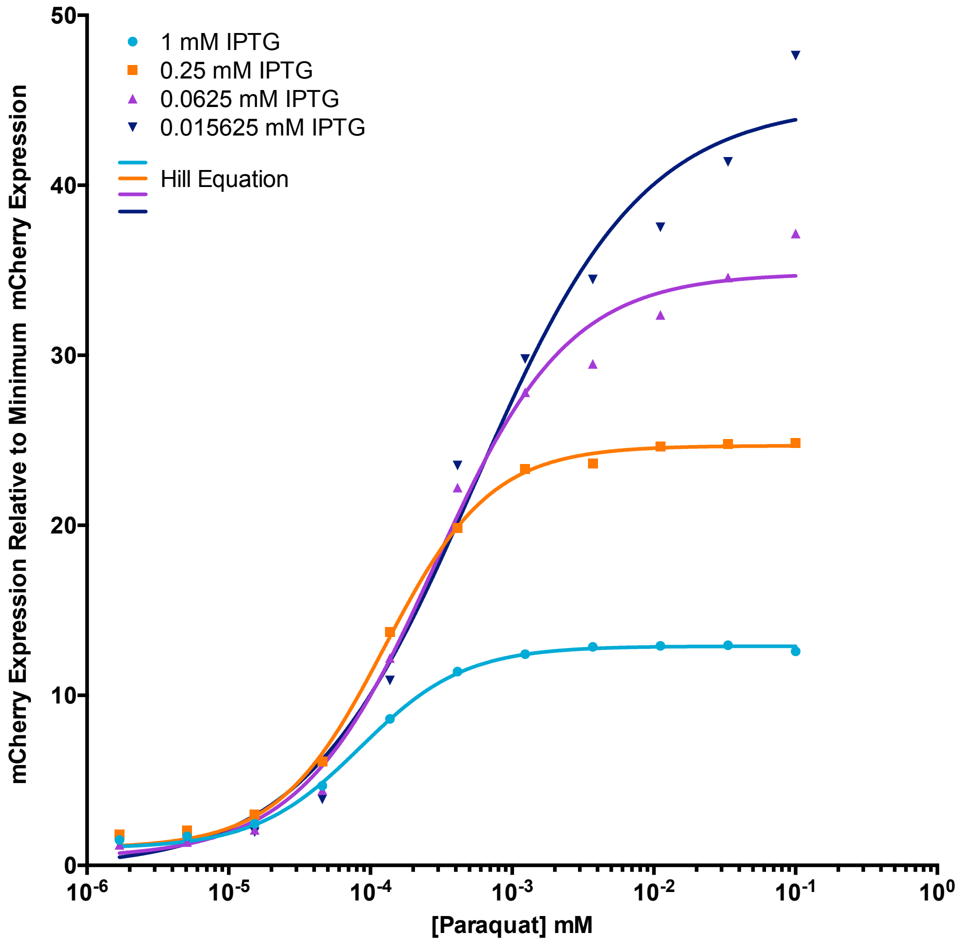


**Supplementary Figure 4 | Normalized mCherry expression for the IPTG-controlled paraquat sensing circuit in Figure 2E.** mCherry expression across a range of paraquat levels was normalized to mCherry expression at zero paraquat for the circuits and data in Figs. 2E and 2F. Lower IPTG concentrations increased the output fold-induction and input dynamic range.

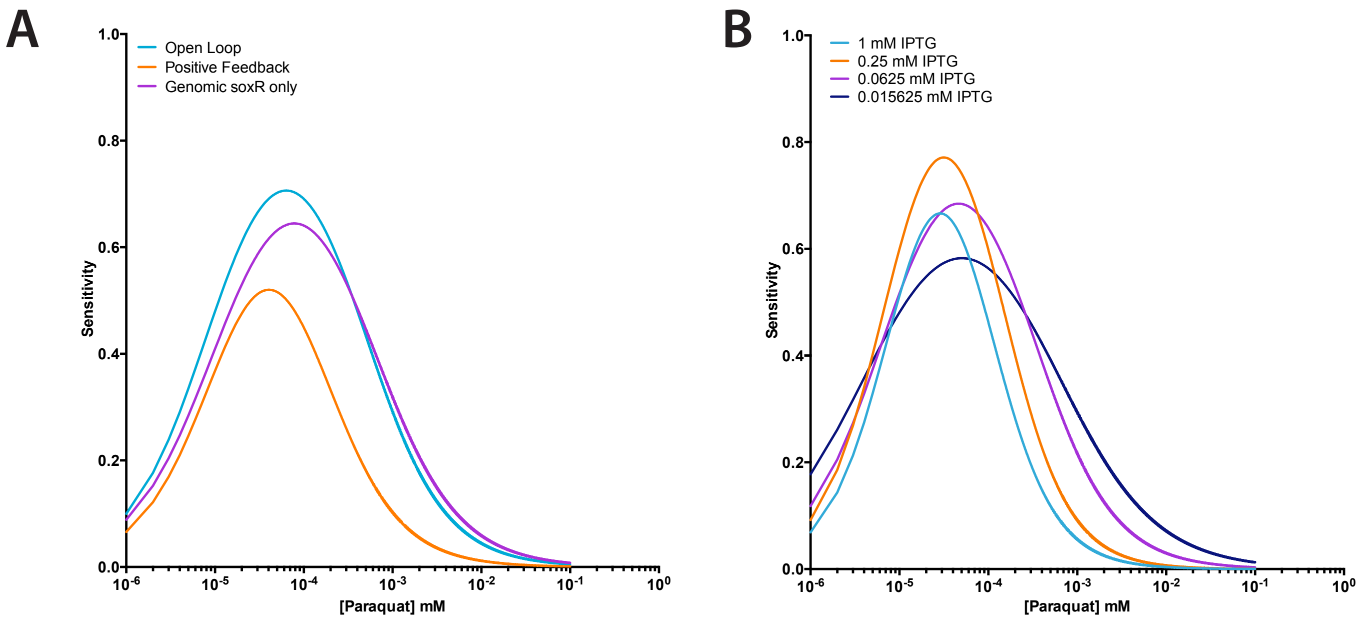


**Supplementary Figure 5 | Sensitivities for different paraquat-sensing circuits. (A)** Sensitivities of the circuits in Figs. 2A, 2B, and Supplementary Fig. 3A, calculated from the Hill curves plotted in Fig. 2C or Supplementary Fig. 3B. **(B)** Sensitivities of the circuits in Fig. 2E, calculated from the Hill curves plotted in Fig. 2F.

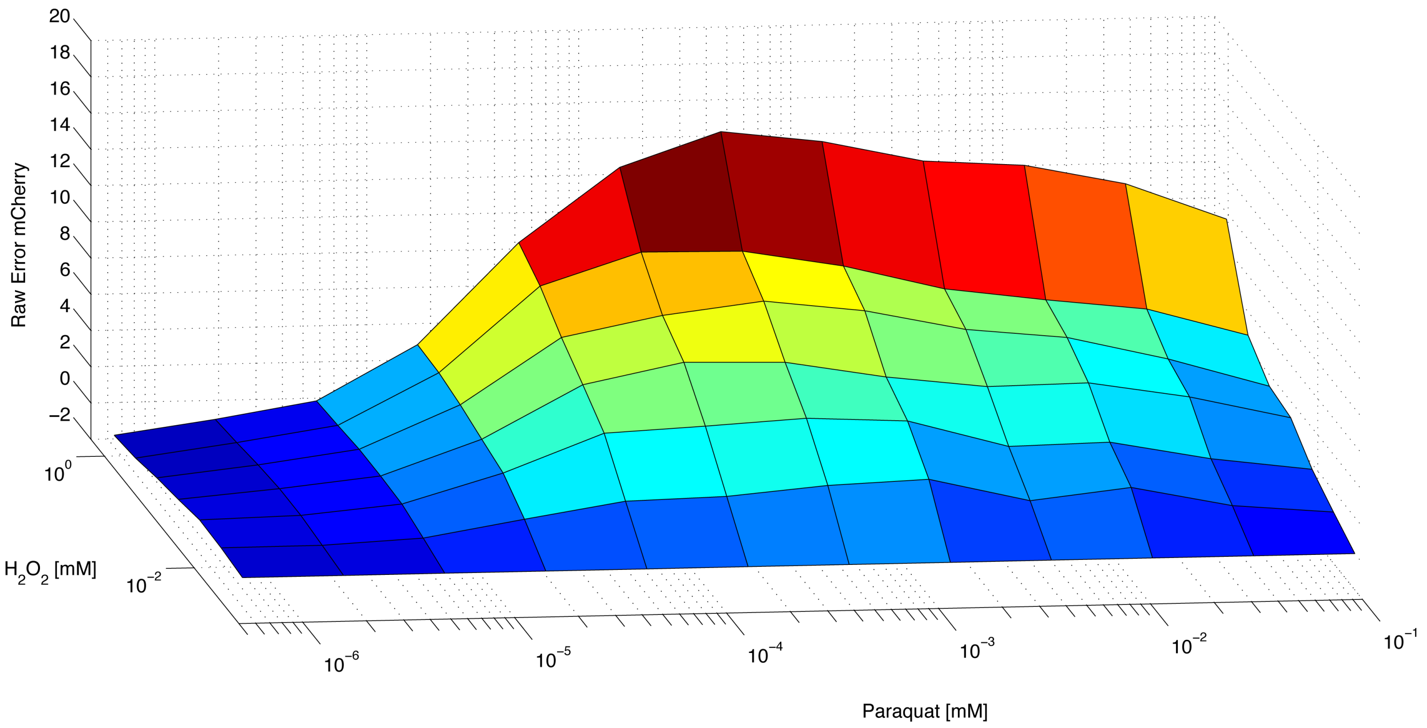


**Supplementary Figure 6 | Raw mCherry error for the first iteration of the dual-sensor circuit (Fig. 3A) based on the data in Fig. 3B.** The data shown is the mean of the raw error calculated from each experimental replicate and is thus different from that shown in Supplementary Note 2. The lowest concentrations of paraquat and $H_2O_2$ tested were zero, but are plotted as non-zero numbers so as to be shown on the logarithmic axes.

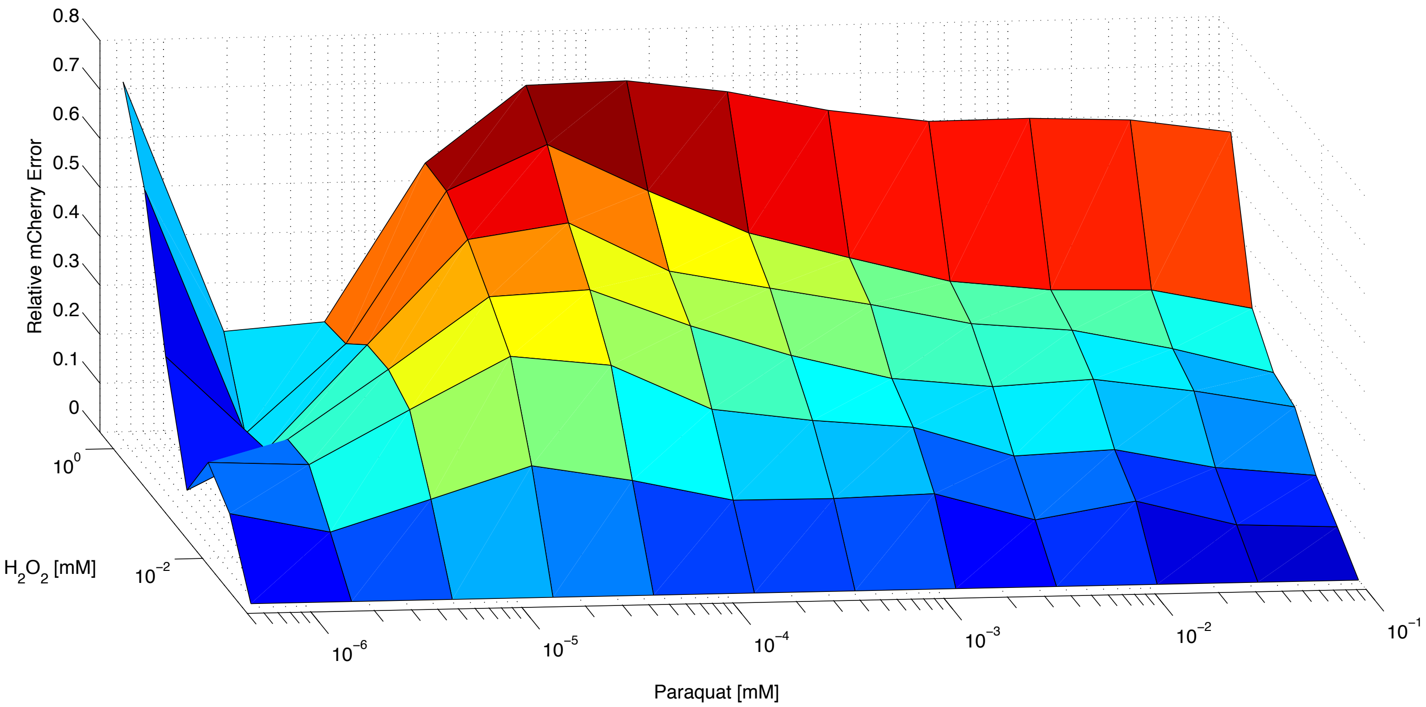


**Supplementary Figure 7 | Relative mCherry error for the first iteration of the dual-sensor circuit (Fig. 3A) based on the data in Fig. 3B.** The data shown is the mean of the relative error calculated from each experimental replicate and is thus different from that shown in Supplementary Note 2. The lowest concentrations of paraquat and $H_2O_2$ tested were zero, but are plotted as non-zero numbers so as to be shown on the logarithmic axes.

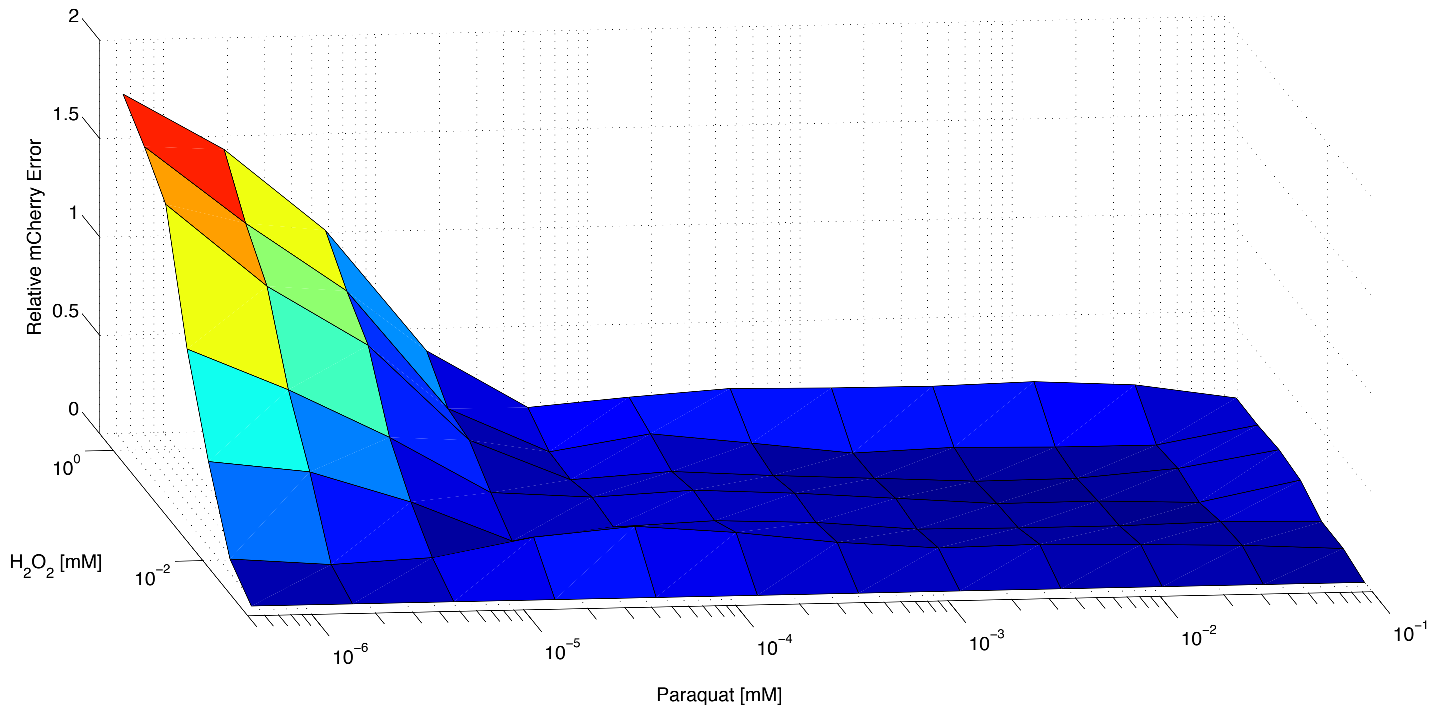


**Supplementary Figure 8 | Relative mCherry error for the dual-sensor circuit with the analog compensation circuit (Fig. 3C) based on the data in Fig. 3D.** Crosstalk is reduced at high concentrations of paraquat, but is higher at low concentrations of paraquat compared to the initial dual-sensing strain (Supplementary Fig. 7). The data shown are the mean of the relative error calculated from each experimental replicate. The lowest concentrations of paraquat and $H_2O_2$ tested were zero, but are plotted as non-zero numbers so as to be shown on the logarithmic axes.

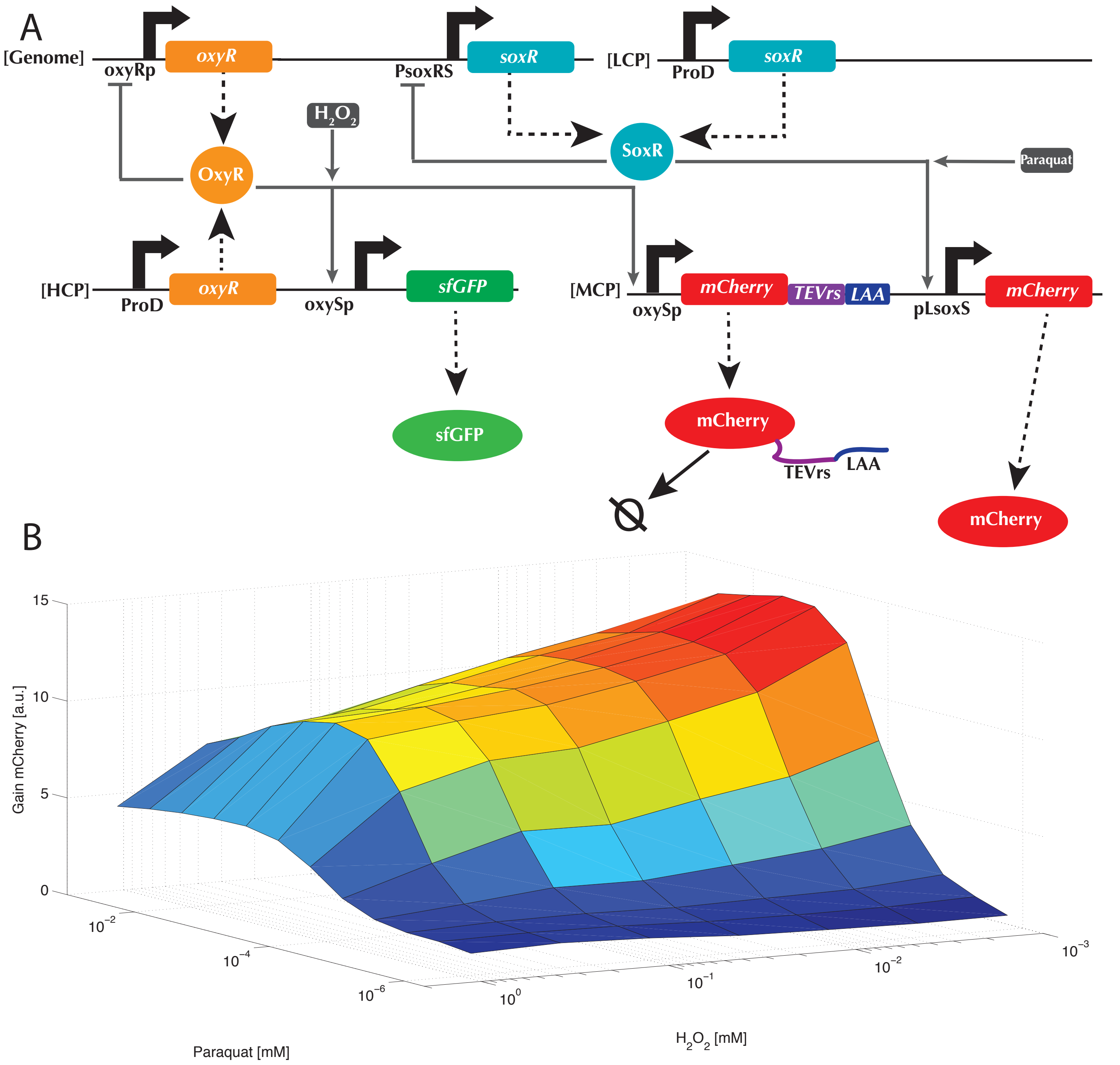


**Supplementary Figure 9 | The dual-sensor circuit with the variable-analog compensation circuit without TEVp. (A)** The dual-sensor circuit with the variable-analog compensation circuit without pLsoxS-*TEVp*. mCherry expressed from oxySp is targeted for degradation due to the LAA degradation signal. **(B)** The mCherry output from the circuit in (A) in terms of fold-change relative to minimum fluorescence. Because oxySp-derived mCherry is rapidly degraded, the mCherry output looks similar to the first iteration of the dual-ROS sensing circuit (Fig. 3B). The data are derived from three biological replicates and flow cytometry experiments, each involving 30,000 events. The lowest concentrations of paraquat and $H_2O_2$ tested were zero, but are plotted as non-zero numbers so as to be shown on the logarithmic axes. Source data are provided as a Source Data file.

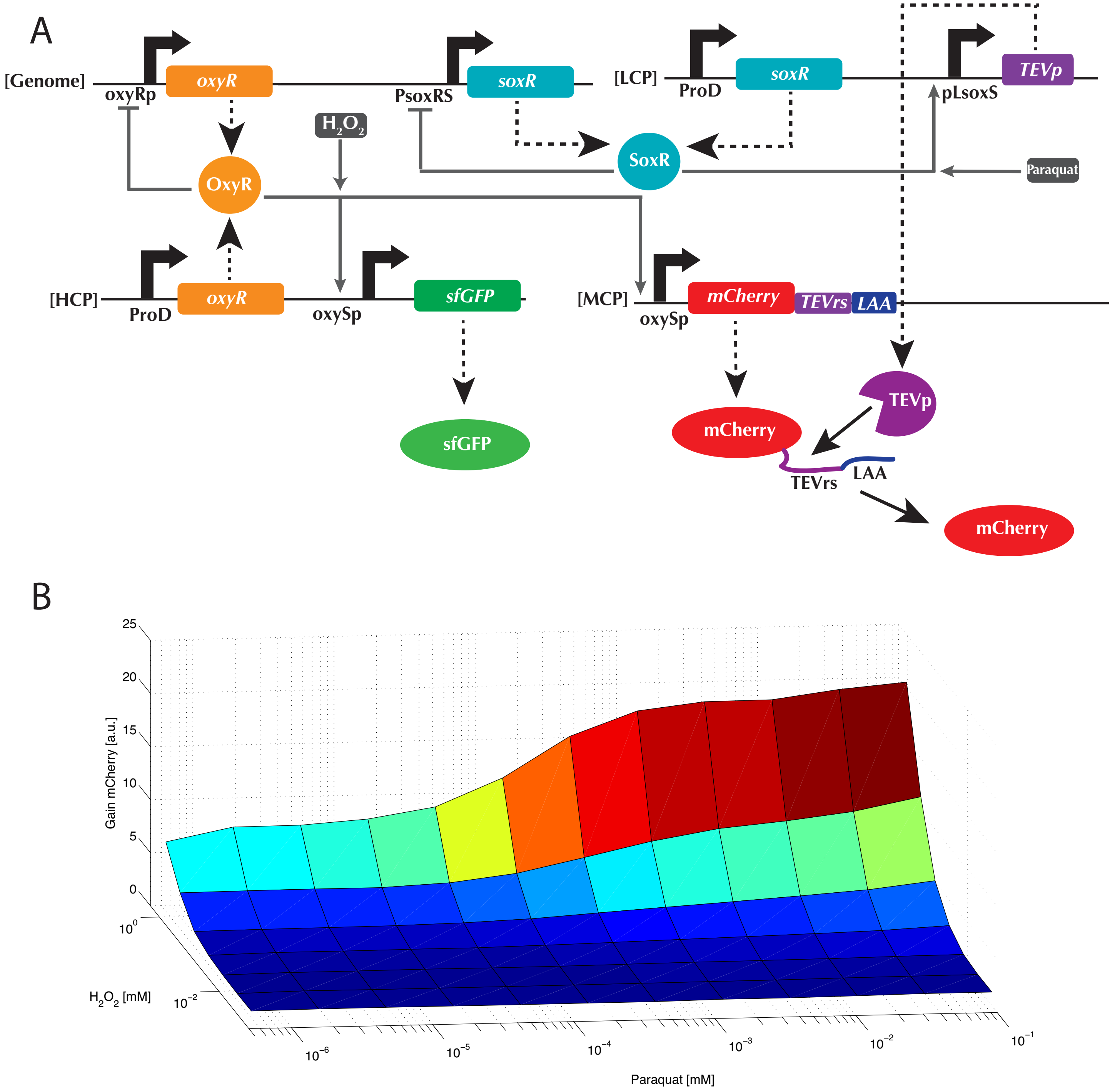
A
[Genome]
oxyRp
oxyR
PsoxRS
soxR
[LCP]
ProD
soxR
pLsoxS
TEVp
$H_2O_2$
OxyR
SoxR
Paraquat
[HCP]
ProD
oxyR
oxySp
sfGFP
[MCP]
oxySp
mCherry
TEVrs
LAA
sfGFP
mCherry
TEVp
TEVrs
LAA
mCherry
B
Gain mCherry [a.u.]
25
20
15
10
5
0
$10^0$
$10^{-2}$
$H_2O_2$ [mM]
$10^{-6}$
$10^{-5}$
$10^{-4}$
$10^{-3}$
$10^{-2}$
$10^{-1}$
Paraquat [mM]

C

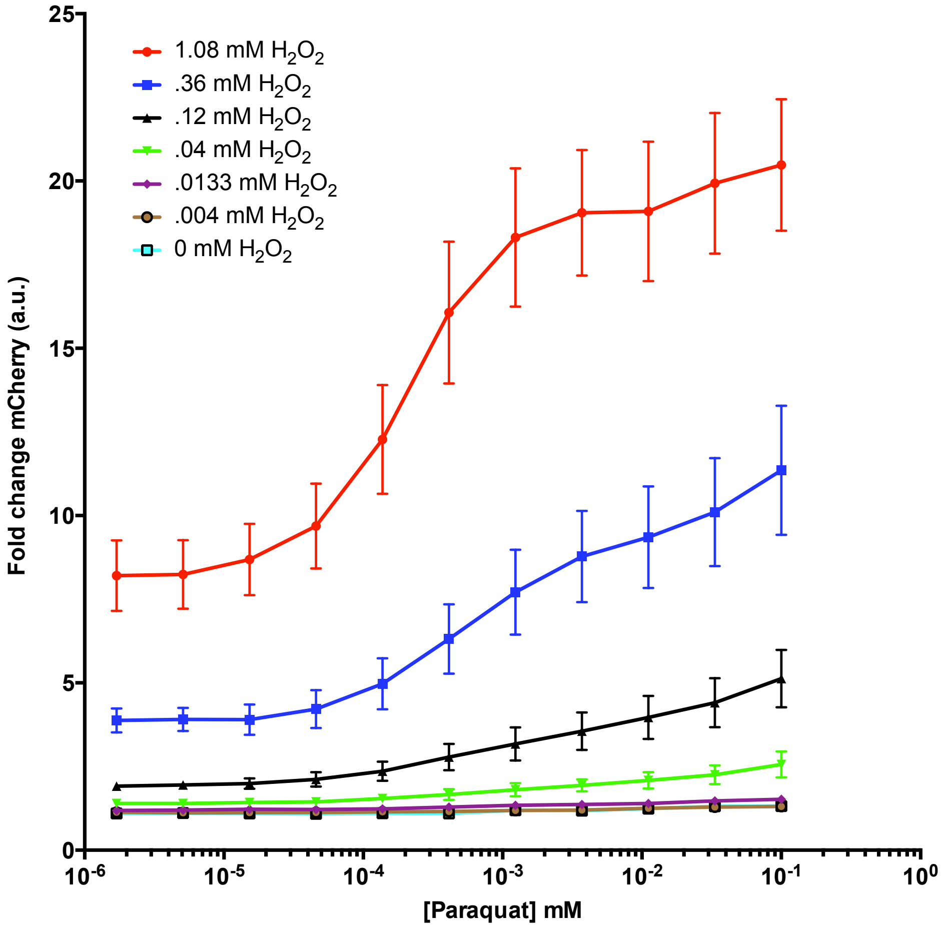


**Supplementary Figure 10 | The dual-sensor circuit with the variable-analog compensation circuit without pLsoxS-mCherry. (A)** In order to characterize the effect of the variable-analog compensation circuit on mCherry expression independently from the SoxR-based system, we removed the pLsoxS-mCherry operon so that mCherry was only expressed from the oxySp promoter. **(B)** The mCherry output in terms of fold-change relative to minimum fluorescence. The "compensation" function of the variable-analog compensation circuit resembles the raw mCherry error for the dual-sensor circuit (Supplementary Fig. 6). The lowest concentrations of paraquat and $H_2O_2$ tested were zero, but are plotted as non-zero numbers so as to be shown on the logarithmic axes. **(C)** The mCherry output from Supplementary Fig. 10B in terms of fold-change of mCherry expression versus paraquat concentration at different concentrations of $H_2O_2$. The errors (s.e.m.) are derived from three biological replicates and flow cytometry experiments, each involving 30,000 events. Source data are provided as a Source Data file.

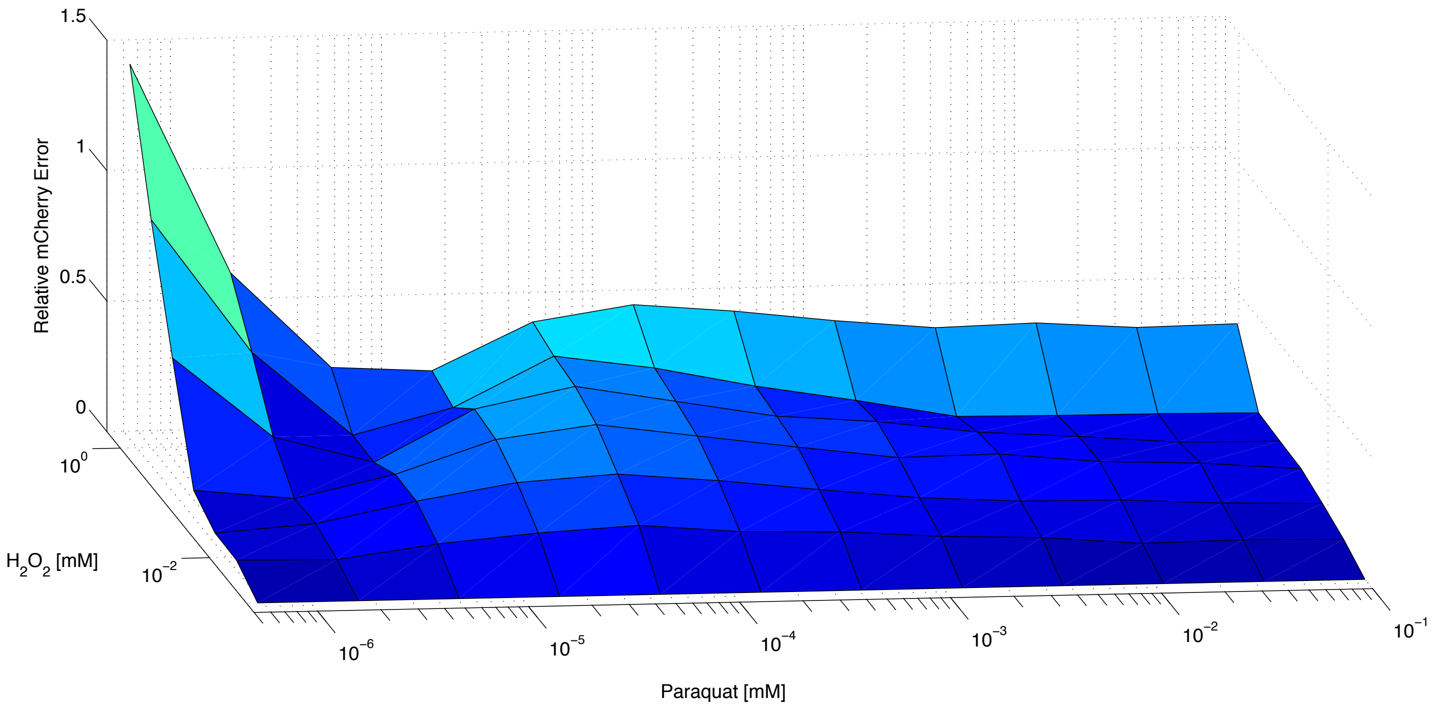


**Supplementary Figure 11 | Relative mCherry error for the dual-sensor circuit with the variable-analog compensation circuit (Fig. 3E) based on the data in Fig. 3F.** Crosstalk is lower at high paraquat levels and is reduced at low paraquat compared to the initial dual-sensor circuit in Supplementary Fig. 7. The data shown is the mean of the relative error calculated from each experimental replicate. The lowest concentrations of paraquat and $H_2O_2$ tested were zero, but are plotted as non-zero numbers so as to be shown on the logarithmic axes.

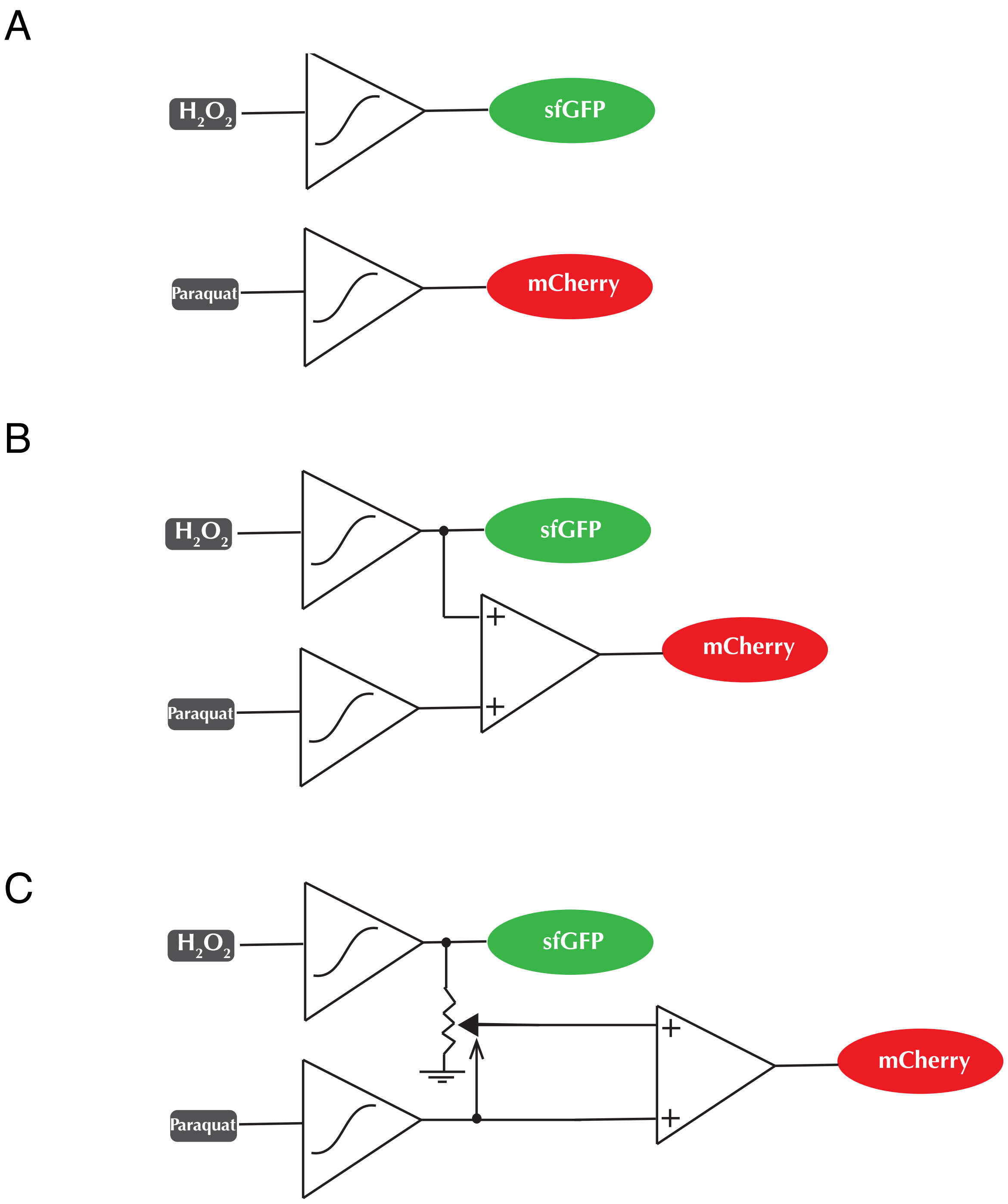


**Supplementary Figure 12 | Abstraction of dual-sensor strains. (A)** The first iteration of the dual-sensor circuit in Fig. 3A. The sfGFP output is dependent upon $H_2O_2$ input concentration and the mCherry output is dependent upon paraquat input concentration. **(B)** The dual-sensor circuit with the analog compensation circuit. The sfGFP output is dependent upon $H_2O_2$ input concentration. The total mCherry output is the sum of mCherry resulting from the $H_2O_2$-sensing circuit and the paraquat-sensing circuit. **(C)** The dual-sensor circuit with the variable-analog compensation circuit. The sfGFP output is dependent upon $H_2O_2$ input concentration. The total mCherry output is the sum of mCherry resulting from the $H_2O_2$-sensing circuit and the paraquat-sensing circuit, where the paraquat-sensing circuit can adjust the amount of mCherry resulting from the $H_2O_2$-sensing circuit by adjusting the mCherry degradation rate. We describe this scenario as being conceptually analogous to a potentiometer whose output can be regulated by a paraquat circuit.

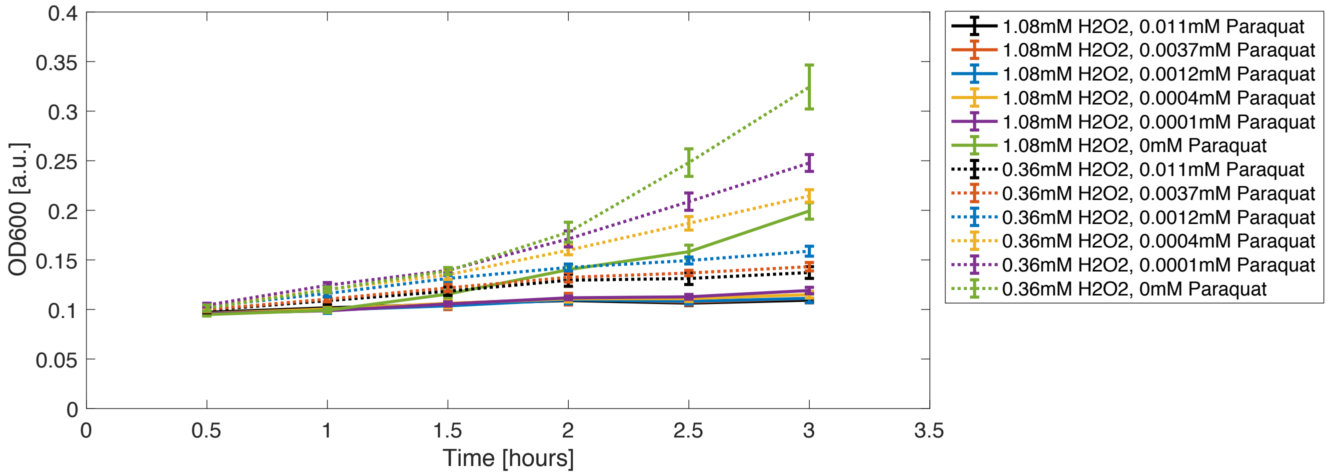


**Supplementary Figure 13 | Growth of the dual-sensor strain in the presence of combinations of high concentrations of ROS.** The dual-sensor strain (Fig. 3A) was exposed to different combinations of high concentrations of paraquat and $H_2O_2$, and growth was assayed every 30 minutes up to 3 hours by measuring the optical density of the culture at 600 nM. 1.08 mM $H_2O_2$ repressed cell growth to a negligible rate in the presence of paraquat. The mean and error (s.d.) shown are calculated from replicates (n=8) derived from the same parent culture. Source data are provided as a Source Data file.

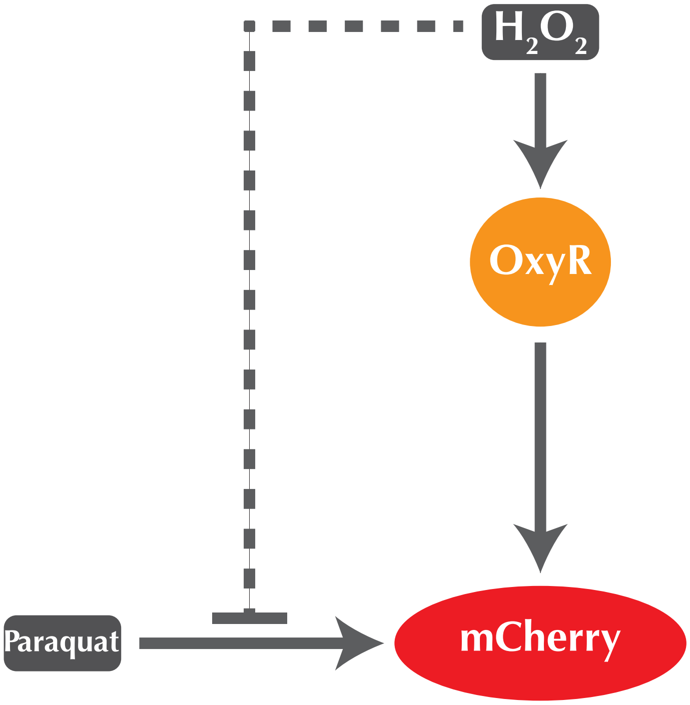


**Supplementary Figure 14 | Incoherent feedforward loop in crosstalk compensation.** $H_2O_2$ represses mCherry expression when paraquat is present via unknown crosstalk mechanisms (dashed line) and activates mCherry expression via a transcriptional connection through OxyR. The net result is that the effect of $H_2O_2$ on mCherry expression is little.

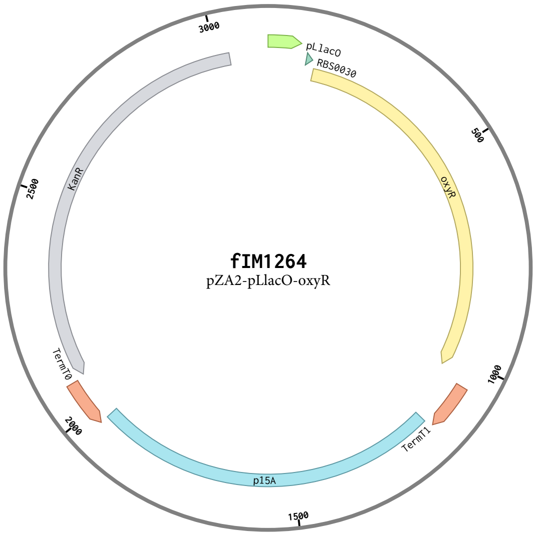
fIM1264
pZA2-pLlacO-oxyR
pLlacO
RBS0030
oxyR
TermT1
p15A
TermT0
KanR
500
1000
1500
2000
2500
3000

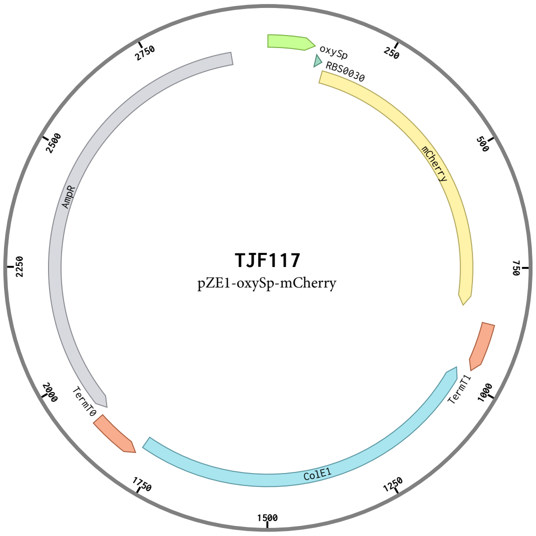
TJF117
pZE1-oxySp-mCherry
oxySp
RBS0030
mCherry
TermT1
ColE1
TermT0
AmpR
250
500
750
1000
1250
1500
1750
2000
2250
2500
2750

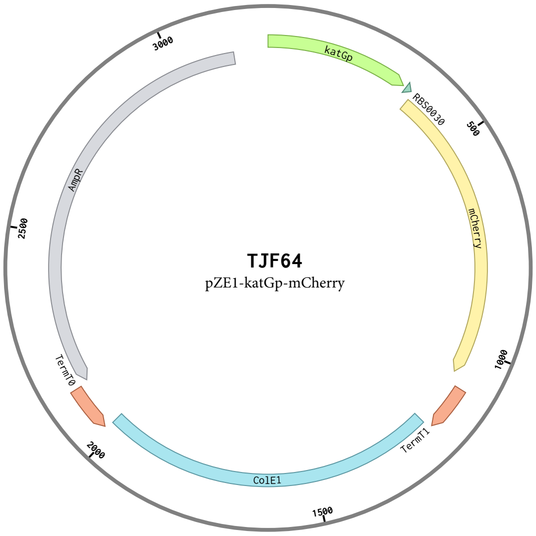
TJF64
pZE1-katGp-mCherry
katGp
RBS0030
mCherry
TermT1
ColE1
TermT0
AmpR
500
1000
1500
2000
2500
3000

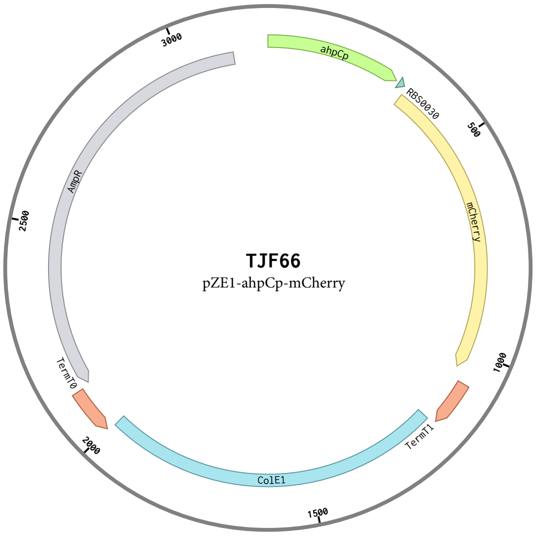
TJF66
pZE1-ahpCp-mCherry
ahpCp
RBS0030
mCherry
TermT1
ColE1
TermT0
AmpR
500
1000
1500
2000
2500
3000

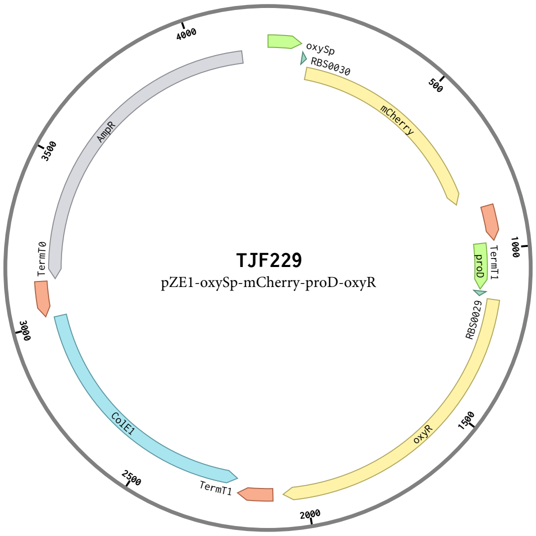
TJF229
pZE1-oxySp-mCherry-proD-oxyR
oxySp
RBS0030
mCherry
TermT1
proD
RBS0029
oxyR
TermT1
ColE1
TermT0
AmpR
500
1000
1500
2000
2500
3000
3500
4000

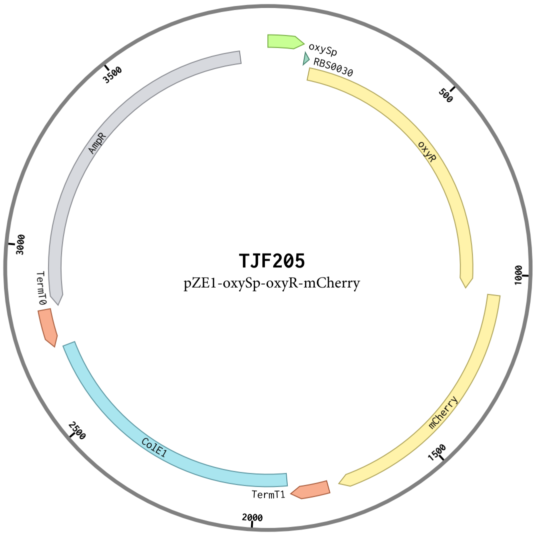
TJF205
pZE1-oxySp-oxyR-mCherry
oxySp
RBS0030
oxyR
mCherry
TermT1
ColE1
TermT0
AmpR
500
1000
1500
2000
2500
3000
3500

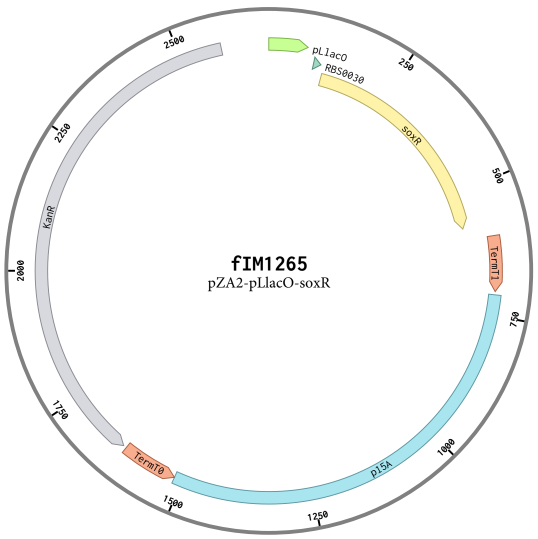
fIM1265
pZA2-pLlacO-soxR
pLlacO
RBS0030
soxR
TermT1
p15A
TermT0
KanR
250
500
750
1000
1250
1500
1750
2000
2250
2500

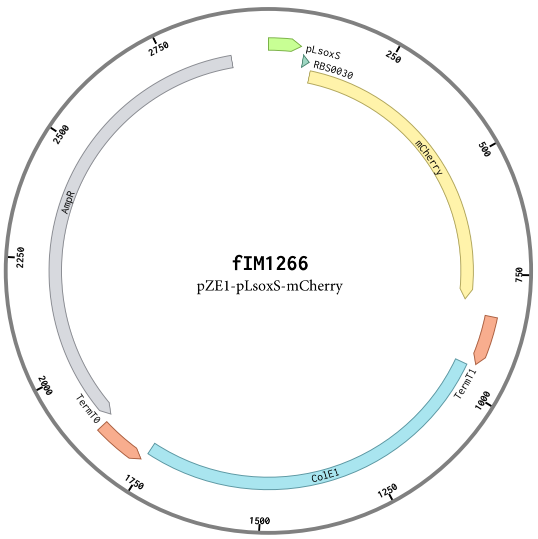
fIM1266
pZE1-pLsoxS-mCherry
pLsoxS
RBS0030
mCherry
TermT1
ColE1
TermT0
AmpR
250
500
750
1000
1250
1500
1750
2000
2250
2500
2750

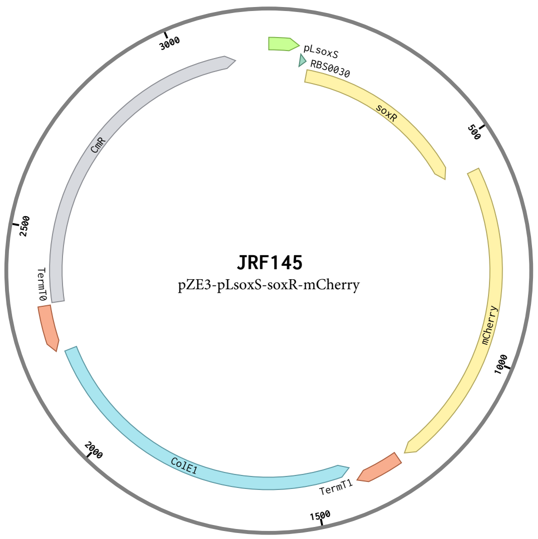
JRF145
pZE3-pLsoxS-soxR-mCherry
pLsoxS
RBS0030
soxR
mCherry
TermT1
ColE1
TermT0
CmR
500
1000
1500
2000
2500
3000

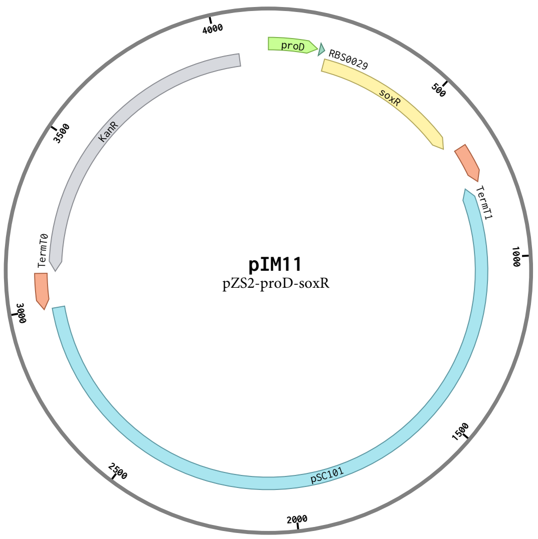
pIM11
pZS2-proD-soxR
proD
RBS0029
soxR
TermT1
pSC101
TermT0
KanR
500
1000
1500
2000
2500
3000
3500
4000

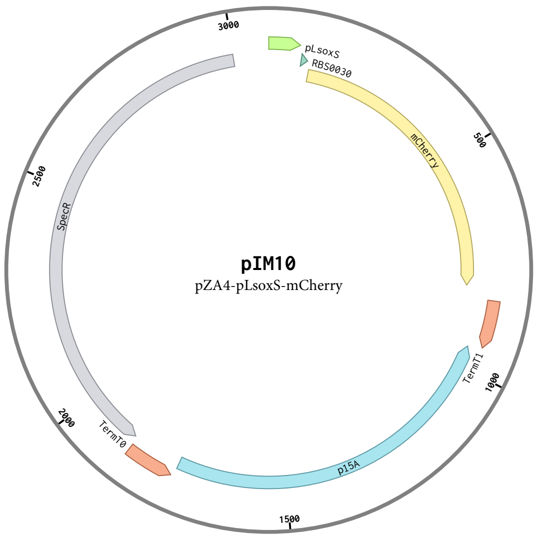
pIM10
pZA4-pLsoxS-mCherry
pLsoxS
RBS0030
mCherry
TermT1
p15A
TermT0
SpecR
500
1000
1500
2000
2500
3000

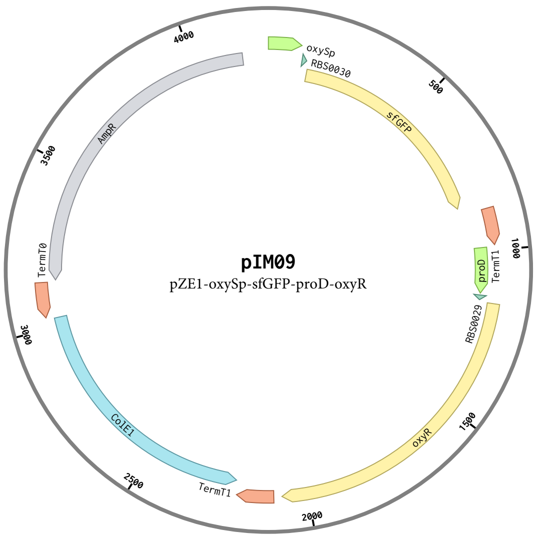
pIM09
pZE1-oxySp-sfGFP-proD-oxyR
oxySp
RBS0030
sfGFP
TermT1
proD
RBS0029
oxyR
TermT1
ColE1
TermT0
AmpR
500
1000
1500
2000
2500
3000
3500
4000

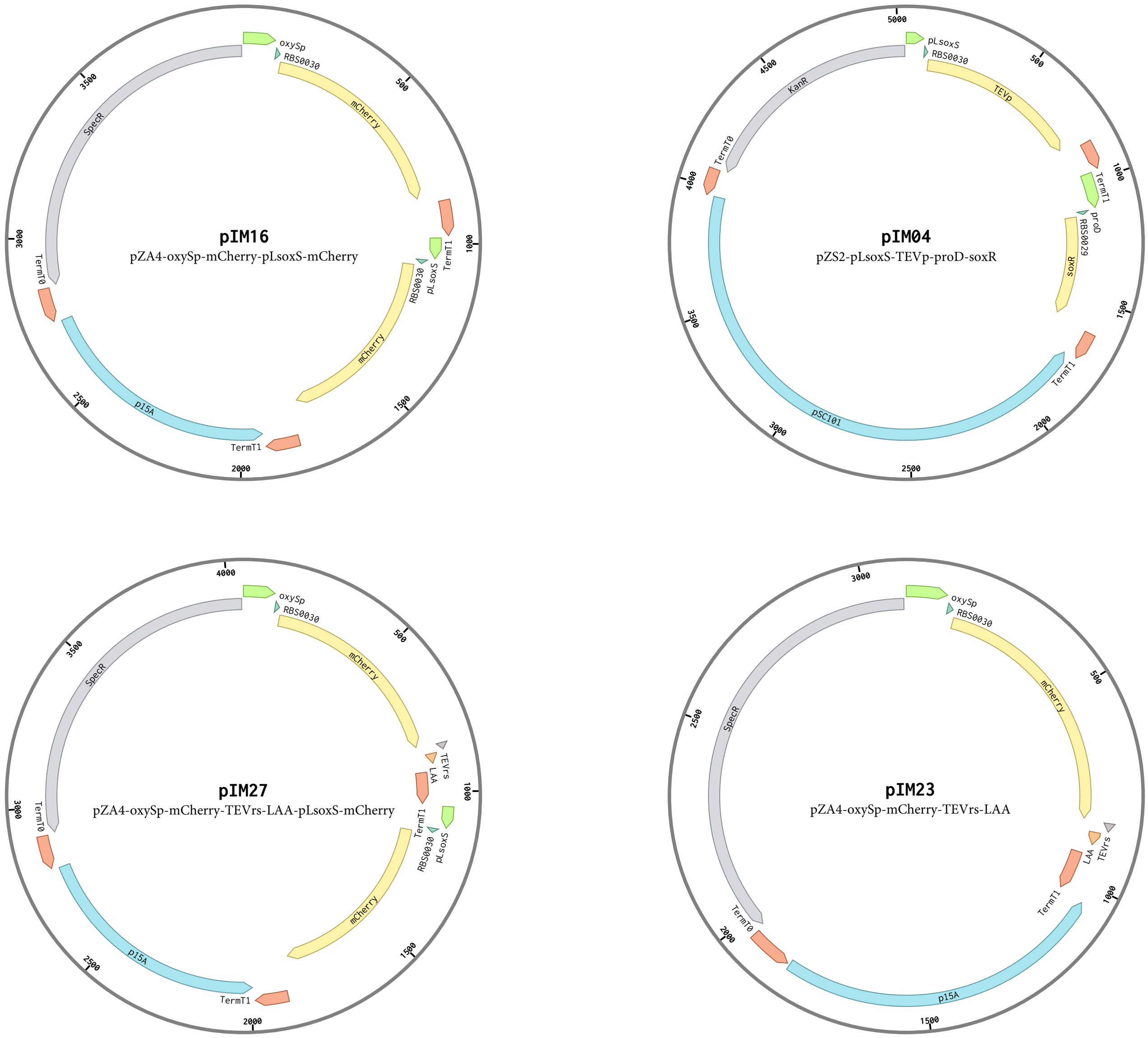


**Supplementary Figure 15 | Plasmid maps used in this study.**

**Supplementary Table 1 | List of plasmids used in experiments**

| Name | Description | Figure |
|---|---|---|
| fIM1264 | pZA2-pLlacO-oxyR | Fig. 1A |
| TJF117 | pZE1-oxySp-mCherry | Fig. 1A |
| TJF64 | pZE1-katGp-mCherry | Fig. 1A |
| TJF66 | pZE1-ahpCp-mCherry | Fig. 1A |
| TJF229 | pZE1-oxySp-mCherry-proD-oxyR | Fig. 1D |
| TJF205 | pZE1-oxySp-oxyR-mCherry | Fig. 1E |
| fIM1265 | pZA2-pLlacO-soxR | Figs. 2A, 2E |
| fIM1266 | pZE1-pLsoxS-mCherry | Figs. 2A, 2E |
| JRF145 | pZE3-pLsoxS-soxR-mCherry | Fig. 2B |
| pIM11 | pZS2-proD-soxR | Figs. 3A, 3C, Supplementary Fig. 9A |
| pIM10 | pZA4-pLsoxS-mCherry | Fig. 3A |
| pIM09 | pZE1-oxySp-sfGFP-proD-oxyR | Figs. 3A, 3C, 3E, Supplementary Figs. 9A, 10A |
| pIM16 | pZA4-oxySp-mCherry-pLsoxS-mCherry | Fig. 3C |
| pIM04 | pZS2-pLsoxS-TEVp-proD-soxR | Fig. 3E, Supplementary Fig. 10A |
| pIM27 | pZA4-oxySp-mCherry-TEVrs-LAA-pLsoxS-mCherry | Fig. 3E, Supplementary Fig. 9A |
| pIM23 | pZA4-oxySp-mCherry-TEVrs-LAA | Supplementary Fig. 10A |

**Supplementary Table 2 | List of synthetic parts**

| **Part Name** | **Description and Source** |
|---|---|
| pLlacO | pLlacO-1 LacI-repressible promoter (3) |
| pLsoxS | The *soxS* promoter SoxR-binding site fused to lambda phage promoter -10 and -35 sequences (4) |
| oxySp | Promoter for *E. coli oxySp* RNA (5) |
| katGp | Promoter for *E. coli katG* gene (5) |
| ahpCp | Promoter for *E. coli ahpC* gene (5) |
| proD | Strong proD promoter (6) |
| RBS0030 | Ribosome binding site. BBa_B0030 (7) |
| RBS0029 | Ribosome binding site. BBa_B0029 (7) |
| *oxyR* | *oxyR* protein-coding sequence (5) |
| *soxR* | *soxR* protein-coding sequence (5) |
| *TEVp* | TEV protease protein-coding sequence (8) |
| *mCherry* | mCherry fluorescent protein coding sequence. BBa_J06504 (7) |
| *sfGFP* | Super-folder green fluorescent protein coding sequence. BBa_I746916 (7) |
| TEVrs | TEV protease recognition sequence (9) |
| LAA | LAA degradation tag (7) |
| TermT1 | Terminator T1 (3) |
| TermT0 | Terminator T0 (3) |
| p15A | Medium-copy number origin of replication (3) |
| ColE1 | High-copy number origin of replication (3) |
| pSC101 | Low-copy number origin of replication (3) |
| *ampR* | Ampicillin-resistance cassette (3) |
| *kanR* | Kanamycin-resistance cassette (3) |
| *specR* | Spectinomycin-resistance cassette (3) |
| *cmR* | Chloramphenicol-resistance cassette (3) |

**Supplementary Note 1: Calculating output fold-induction, relative input range, sensitivity, and utility**

1. Calculate best-fitting Hill function from the raw data:

   Hill functions are of the form:

$$y = \frac{b_{max} * x^n}{K_d{}^n + x^n} + C$$

   Where $C$ is the empirical geometric mean (y) at zero input (x = 0), and $n$, $K_d$, and $b_{max}$ are fit to the data.

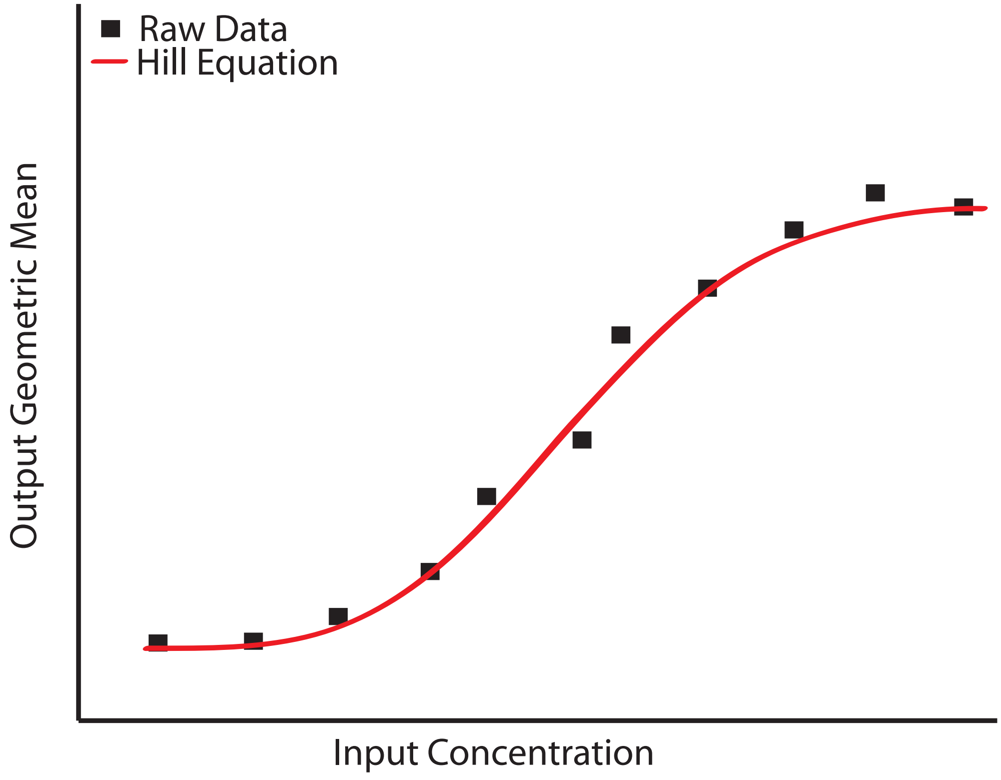


2. Calculate the output fold-induction (FI):

$$FI = \frac{(b_{max} + C)}{C}$$

   If the observed maximum gene expression is less than the theoretical maximum gene expression ($b_{max} + C$), then the observed maximum gene expression ($b_{max,\ observed}$) is used to calculate the output fold-induction. In this case, the output fold-induction is:

$$FI = \frac{(b_{max,observed})}{C}$$

3. Calculate the Input Dynamic Range (*10*):

**A)** In the case where the theoretical $b_{max}$ is less than the maximum gene expression observed:

90% of maximum output ($Y_{90}$) is calculated as:

$$Y_{90} = C + .9 * b_{max}$$

10% of maximum output ($Y_{10}$) is calculated as:

$$Y_{10} = C + .1 * b_{max}$$

$Y_{90}$ and $Y_{10}$ are interpolated to the X-axis using the Hill function to determine $X_{90}$ and $X_{10}$.

The input concentration for 90% of the maximum output ($X_{90}$) is calculated as:

$$X_{90} = K_d * \sqrt[n]{9}$$

The input concentration for 10% of the maximum output ($X_{10}$) is calculated as:

$$X_{10} = \frac{K_d}{\sqrt[n]{9}}$$

The relative input range (RIR) is calculated as:

$$RIR = \frac{X_{90}}{X_{10}} = \sqrt[n]{81}$$

**B)** In the case where the theoretical $b_{max}$ is greater than the max gene expression observed:

90% of maximum output ($Y_{90}$) is calculated as:

$$Y_{90} = C + .9 * (b_{max,observed} - C)$$

10% of maximum output ($Y_{10}$) is calculated as:

$$Y_{10} = C + .1 * (b_{max,observed} - C)$$

$Y_{90}$ and $Y_{10}$ are interpolated to the X-axis using the Hill function to determine $X_{90}$ and $X_{10}$.

The input concentration for 90% of the maximum output ($X_{90}$) is calculated as:

$$X_{90} = K_d * \sqrt[n]{\frac{b_{max,observed} - C}{\frac{10}{9} * b_{max} + C - b_{max,observed}}}$$

The input concentration for 10% of the maximum output ($X_{10}$) is calculated as:

$$X_{10} = K_d * \sqrt[n]{\frac{b_{max,observed} - C}{10 * b_{max} + C - b_{max,observed}}}$$

The relative input range (RIR) is calculated as:

$$RIR = \frac{X_{90}}{X_{10}} = \sqrt[n]{\frac{10 * b_{max} + C - b_{max,observed}}{\frac{10}{9} * b_{max} + C - b_{max,observed}}}$$

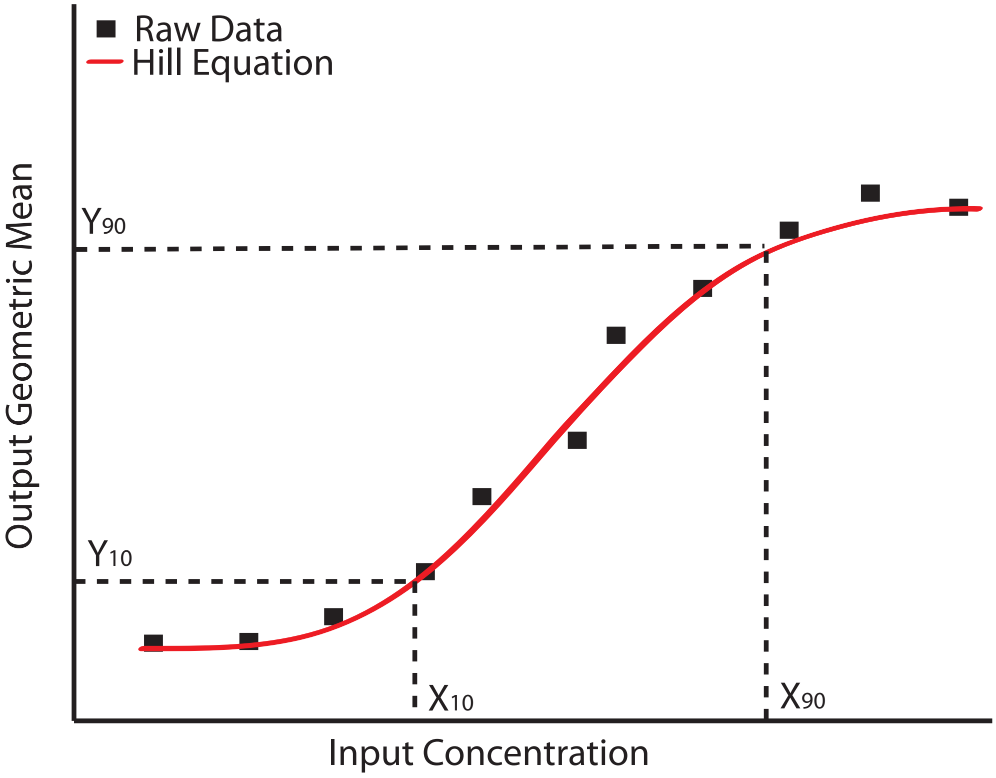


4. Calculate utility by multiplying the relative input dynamic range by the output fold-induction:

$$Utility = FI * RIR$$

**Supplementary Note 2: Calculating errors due to crosstalk between circuits**

1. Calculate the raw error:

   In the ideal scenario for the circuits from Fig. 3, mCherry expression should only be controlled by the input paraquat concentration. To determine the raw error in mCherry expression, we subtracted the mCherry level at every given concentration of paraquat and $H_2O_2$ from the mCherry level at the same concentration of paraquat but with zero $H_2O_2$.

   $$Raw\ error = GeneExpression_{paraquat_a,\ H_2O_{2\ b}} - GeneExpression_{paraquat_a,\ H_2O_{2\ 0}}$$

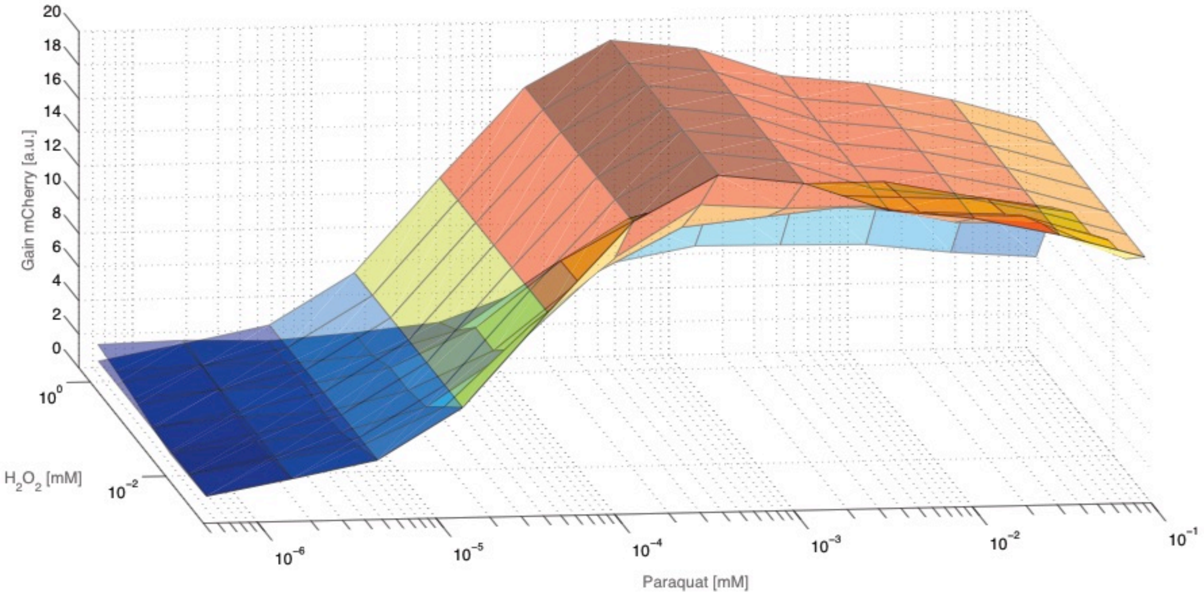


   In the figure above, mCherry expression across a range of paraquat concentrations and zero $H_2O_2$ is extrapolated and overlaid on top of mCherry expression across the entire range of paraquat and $H_2O_2$ concentrations. The difference between these two mCherry levels is the raw error. The raw error for a single experimental replicate for the circuit in Fig. 3A is shown below. The lowest concentrations of paraquat and $H_2O_2$ tested were zero, but are plotted as non-zero numbers so as to be shown on the logarithmic axes.

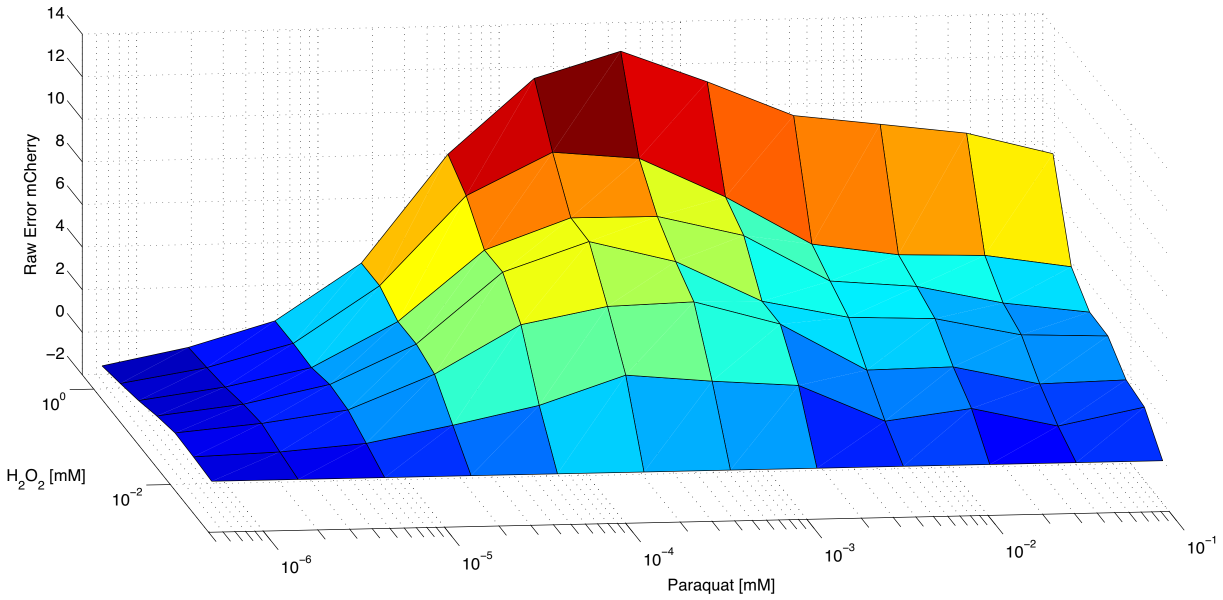

2. Calculate the absolute error:

The absolute error is calculated by taking the absolute value of the raw error. The absolute error for a single experimental replicate for the circuit in Fig. 3A is shown below. The lowest concentrations of paraquat and $H_2O_2$ tested were zero, but are plotted as non-zero numbers so as to be shown on the logarithmic axes.

$$Absolute\ error = \left|GeneExpression_{paraquat_a,\ H_2O_{2\ b}} - GeneExpression_{paraquat_a,\ H_2O_{2\ 0}}\right|$$

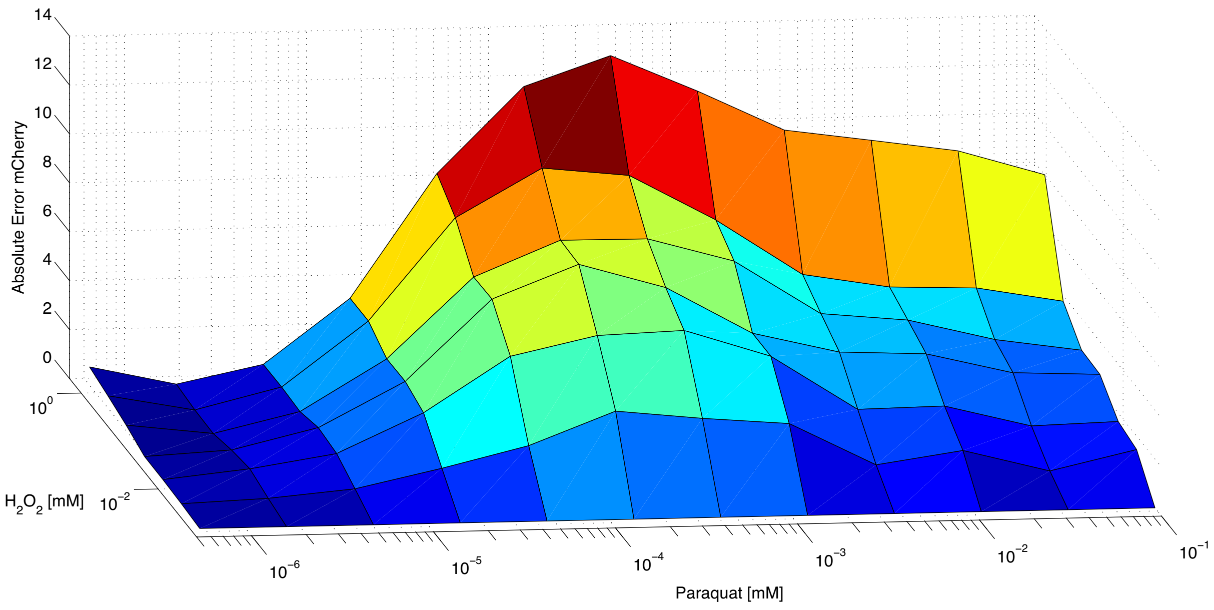


3. Calculate the relative error:

The relative error is calculated by normalizing the absolute error to the corresponding mCherry level at the same concentration of paraquat but with zero $H_2O_2$. The relative error for an experimental replicate for the circuit in Fig. 3A is shown below. The lowest concentrations of paraquat and $H_2O_2$ tested were zero, but are plotted as non-zero numbers so as to be shown on the logarithmic axes.

$$Relative\ error = \frac{\left|GeneExpression_{paraquat_a,\ H_2O_{2\ b}} - GeneExpression_{paraquat_a,\ H_2O_{2\ 0}}\right|}{GeneExpression_{paraquat_a,\ H_2O_{2\ 0}}}$$

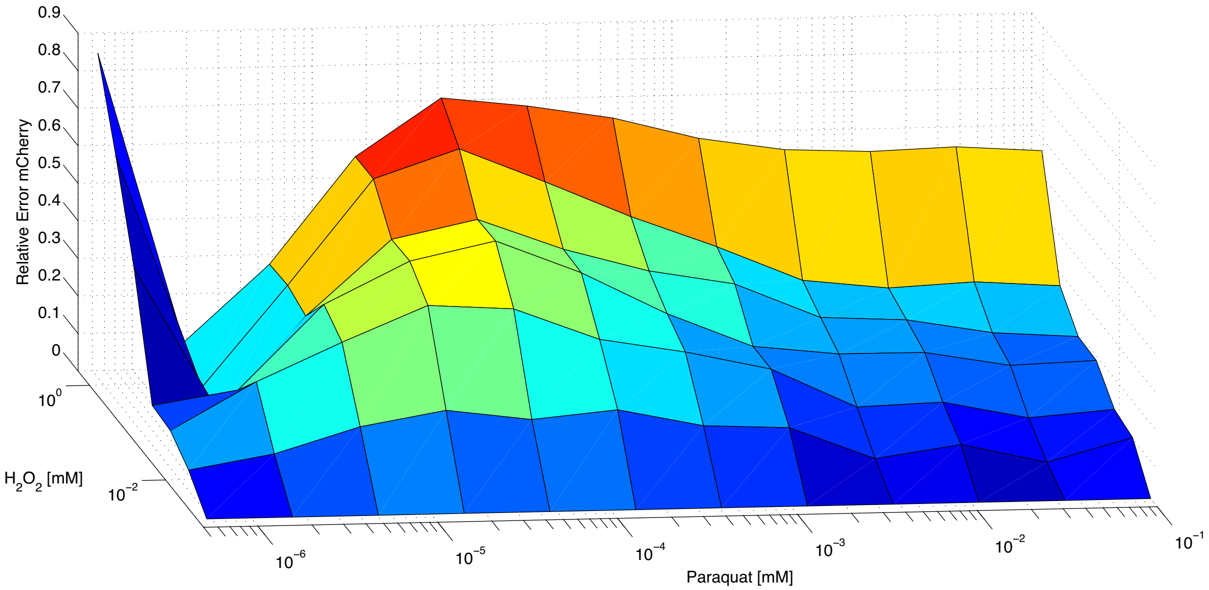


4. Sum the relative errors to get the total relative error:

   To calculate the total relative error, the relative error at every concentration of paraquat and $H_2O_2$ is summed.

$$Total\ relative\ error = \sum_{paraquat_0,\ H_2O_{2\ 0}}^{paraquat_{max},\ H_2O_{2\ max}} \frac{\left| GeneExpression_{paraquat_a,\ H_2O_{2\ b}} - GeneExpression_{paraquat_a,\ H_2O_{2\ 0}} \right|}{GeneExpression_{paraquat_a,\ H_2O_{2\ 0}}}$$

This procedure for calculating errors has been illustrated for the mCherry output of a single experimental replicate of the circuit shown in Fig. 3A. The analogous procedure can be carried out for the sfGFP output of the circuits in Fig. 3, thus resulting in the calculations plotted in Fig. 3I.

## Supplementary References


1. Shibata, T. & Fujimoto, K. Noisy signal amplification in ultrasensitive signal transduction. *Proc. Natl. Acad. Sci. USA* **102**, 331-336 (2005).
2. Hidalgo, E., Leautaud, V. & Demple, B. The redox-regulated SoxR protein acts from a single DNA site as a repressor and an allosteric activator. *EMBO J.* **17**, 2629-2636 (1998).
3. Lutz, R. & Bujard, H. Independent and tight regulation of transcriptional units in *Escherichia coli* via the LacR/O, the TetR/O and AraC/$I_1$-$I_2$ regulatory elements. *Nucleic Acids Res.* **25**, 1203-1210 (1997).
4. Dwyer, D. J., Kohanski, M. A., Hayete, B. & Collins, J. J. Gyrase inhibitors induce an oxidative damage cellular death pathway in *Escherichia coli*. *Mol. Syst. Biol.* **3**, 91 (2007).
5. The EcoCyc Database, (available at http://ecocyc.org/).
6. Davis, J. H., Rubin, A. J. & Sauer, R. T. Design, construction and characterization of a set of insulated bacterial promoters. *Nucleic Acids Res.* **39**, 1131-1141 (2011).
7. Registry of Standard Biological Parts, (available at http://igem.org/registry).
8. Kapust R. B. et al. Tobacco etch virus protease: mechanism of autolysis and rational design of stable mutants with wild-type catalytic proficiency. *Protein Eng.* **14**, 993-1000 (2001).
9. Kostallas, G., Löfdahl, P. Å. & Samuelson, P. Substrate Profiling of Tobacco Etch Virus Protease Using a Novel Fluorescence-Assisted Whole-Cell Assay. *PLoS ONE* **6**, e16136 (2011).
10. Madar, D., Dekel, E., Bren, A. & Alon, U. Negative auto-regulation increases the input dynamic-range of the arabinose system of *Escherichia coli*. *BMC Syst. Biol.* **5**, 111 (2011).
